# Correction: Gene expression and chromatin conformation of microglia in virally suppressed people with HIV

**DOI:** 10.26508/lsa.202403085

**Published:** 2024-10-28

**Authors:** Johannes CM Schlachetzki, Sara Gianella, Zhengyu Ouyang, Addison J Lana, Xiaoxu Yang, Sydney O’Brien, Jean F Challacombe, Peter J Gaskill, Kelly L Jordan-Sciutto, Antoine Chaillon, David Moore, Cristian L Achim, Ronald J Ellis, Davey M Smith, Christopher K Glass

**Affiliations:** 1 Department of Cellular and Molecular Medicine, University of California San Diego , San Diego, CA, USA; 2 Department of Neurosciences, University of California San Diego , San Diego, CA, USA; 3 Department of Medicine, Division of Infectious Diseases and Global Public Health, University of California San Diego , San Diego, CA, USA; 4 Department of Human Genetics, University of Utah, Salt Lake City, UT, USA; 5 Department of Pharmacology and Physiology, Drexel University College of Medicine, Philadelphia, PA, USA; 6 Department of Oral Medicine, School of Dental Medicine, University of Pennsylvania, Philadelphia, PA, USA; 7 Department of Psychiatry, University of California San Diego , San Diego, CA, USA; 8 Department of Pathology, University of California San Diego , San Diego, CA, USA

## Abstract

“Despite ART, we detected occasional microglia containing cell-associated HIV RNA and HIV DNA integrated into open regions of the host’s genome (∼0.005%)” should be corrected to: “Despite ART, we detected occasional microglia containing cell-associated HIV RNA and HIV DNA integrated into open regions of the host’s genome (∼0.5%).”

## Abstract (precis)

Leveraging our rapid autopsy “Last Gift” cohort at UCSD, we identified distinct microglial gene expression profiles despite ART.

Article: Schlachetzki JCM, Gianella S, Ouyang Z, Lana AJ, Yang X, O’Brien S, Challacombe JF, Gaskill PJ, Jordan-Sciutto KL, Chaillon A, Moore D, Achim CL, Ellis RJ, Smith DM, Glass CK (2024 Jul 26) Gene expression and chromatin conformation of microglia in virally suppressed people with HIV. Life Sci Alliance 7(10): e202402736. doi: https://doi.org/10.26508/lsa.202402736. PMID: 39060113.

Regarding our article “Gene expression and chromatin conformation of microglia in virally suppressed people with HIV,” we would like to correct errors in the representation of the data concerning the fraction of infected cells. The data presented as percentages in the article were fractions in the original article. For example, HIV RNA was detected in 107 out of 25,091 cells. The fraction is 0.0043. However, we wrote 0.0043%, which should have been 0.43%. We now present the data as percentages.

## Results

In the paragraph titled “HIV RNA and HIV DNA detection in brain myeloid cells,” the following revisions should be made:

 (1) HIV RNA was detected in 107 out of 25,091 CD45^+^ cells (**0.43%**).
 (2) The number of reads mapping to the HIV reference genome ranged between 1 and 270 per cell. LG14, the individual with the highest levels of HIV DNA from bulk DLPFC based on ddPCR, also showed the most HIV RNA^+^ cells with 82 cells out of 6,014 sequenced cells or 1.36%.
 (3) LG15 had 25 cells with detectable HIV RNA out of 11,978 sequenced cells or **0.2%**.
 (4) In line with the results from scRNA-seq, LG14 had with 64 the greatest number of cells with detectable HIV DNA in open chromatin states, while 16 microglia isolated from LG15 had detectable HIV DNA by ATAC-seq (**0.54%** and **0.18%**, respectively).

## Discussion

 (1) “We detected reads of HIV RNA in ∼0.005% of isolated microglia.” should be corrected to “We detected reads of HIV RNA in ∼0.5% of isolated microglia.”
 (2) “The small number of infected cells here is similar to previous studies focusing on the T cell compartment.” should be corrected to “The small number of infected cells here is higher than previous studies focusing on the T cell compartment.”

